# Influence of sub-inhibitory concentrations of antimicrobials on micrococcal nuclease and biofilm formation in *Staphylococcus aureus*

**DOI:** 10.1038/s41598-021-92619-9

**Published:** 2021-06-24

**Authors:** Colin W. K. Rosman, Henny C. van der Mei, Jelmer Sjollema

**Affiliations:** University of Groningen, University Medical Center Groningen, Department of Biomedical Engineering, Groningen, The Netherlands

**Keywords:** Antimicrobials, Applied microbiology, Bacteria, Biofilms

## Abstract

A major contributor to biomaterial associated infection (BAI) is *Staphylococcus aureus*. This pathogen produces a protective biofilm, making eradication difficult. Biofilms are composed of bacteria encapsulated in a matrix of extracellular polymeric substances (EPS) comprising polysaccharides, proteins and extracellular DNA (eDNA). *S. aureus* also produces micrococcal nuclease (MN), an endonuclease which contributes to biofilm composition and dispersion, mainly expressed by *nuc1*. MN expression can be modulated by sub-minimum inhibitory concentrations of antimicrobials. We investigated the relation between the biofilm and MN expression and the impact of the application of antimicrobial pressure on this relation. Planktonic and biofilm cultures of three *S. aureus* strains, including a *nuc1* deficient strain, were cultured under antimicrobial pressure. Results do not confirm earlier findings that MN directly influences total biomass of the biofilm but indicated that *nuc1* deletion stimulates the polysaccharide production per CFU in the biofilm in in vitro biofilms. Though antimicrobial pressure of certain antibiotics resulted in significantly increased quantities of polysaccharides per CFU, this did not coincide with significantly reduced MN activity. Erythromycin and resveratrol significantly reduced MN production per CFU but did not affect total biomass or biomass/CFU. Reduction of MN production may assist in the eradication of biofilms by the host immune system in clinical situations.

## Introduction

An increasing number of biomedical implants is being used in clinical practice. This is due to technical advancement, innovative therapies, increased patient demands and life expectancy. These implants come with the risk of biomaterial associated infection (BAI)^1^. Simultaneously the rate of BAI per implant is increasing due to increasing age and comorbidities of the patients receiving such implants^2, 3^. Treatment of BAI often entails revision of the implant and antibiotic therapy with a risk of secondary infection^4^. This results in increased morbidity and high healthcare costs^1^. Primary antibiotic therapy is usually ineffective since bacteria create a protective shelter when attached to the surface of the foreign material, also designated as a ‘biofilm’. Biofilms are composed of bacteria encapsulated in a matrix of extracellular polymeric substances (EPS) providing protection from antibiotics, the host immune system, and physical and mechanical stress^5^. The EPS content includes polysaccharide intercellular adhesin (PIA), proteins and extracellular DNA (eDNA), all playing a major role in biofilm maturation and structure^6, 7^. eDNA in particular has a glue-like function by keeping bacteria entangled within the biofilm by electrostatic and acid–base interactions with cell surfaces and polysaccharides^7, 8^.

One of the most prominent pathogens in BAI is *Staphylococcus aureus*, a strong biofilm producer responsible for 34% of all orthopedic BAI^9^. An important virulence factor of *S. aureus* is micrococcal nuclease (MN), a thermostable endonuclease that degrades eDNA as a constituent of the biofilm^10, 11^. Biofilm formation and maturation is expected to be intrinsically affected by the production of MN since it cleaves eDNA. MN production is largely regulated by the *SaeRS* gene system^10, 12^. *SaeRS* consists of a sensory SaeS part and a SaeR response regulator and is activated mainly in the post-exponential growth phase by phagocytosis-related signals, sub-minimum inhibitory concentrations (sub-MIC) of some antibiotics and certain chemical stimuli^13^. *S. aureus* produces two types of MN. The first is secreted by the bacterium and encoded by the *nuc1* gene. The second is cell-wall bound and encoded by *nuc2*^10, 14^. *Nuc1* and *nuc2* are expressed in different ratios depending on the growth phase, with *nuc1* being expressed mainly in the post-exponential phase and *nuc2* in the early-exponential phase^10, 15^. In post-exponential cultures of *S. aureus nuc2* accounts for a minimal part of the DNA degrading capabilities of *S. aureus* cultures^15^. Our previous research estimated that about 1% of the nuclease activity at the post-exponential phase can be contributed to *nuc2*, as nuclease activity is decreased by 99% when comparing a *S. aureus* Newman and its *nuc1* deficient mutant^16^. Controversy exists about the effect of MN on biofilm formation. A negative correlation was observed in vitro between biofilm biomass and the nuclease activity in biofilms of various *S. aureus* strains and *nuc1* mutants^17^, whereas in an in vitro catheter model no effect was found on biofilm formation in a *nuc1* and *nuc2* mutant of *S. aureus* UAMS-1 strains^18^.

MN expression and in particular the *SaeRS* system can be modulated by sub-minimum inhibitory concentrations of antimicrobials (see also Table 1)^19, 20^. Vice versa, *SaeRS* may also regulate the *AtlA* autolysin gene, responsible for programmed cell lysis and eDNA release^21, 22^, which makes the production of nuclease and release of e-DNA delicately balanced processes.

**Table 1:** Antimicrobials involved in this study, including working mechanism and effect on bacteria at sub-inhibitory concentration.

Antimicrobial (group)	Bacteriostatic/bactericidal	Bacterial substrate	Sub-MIC effect
Ciprofloxacin (fluoroquinolone)	Bactericidal	Inhibition DNA-gyrase	Increased expression alpha hemolysin and fibronectin binding protein^20, 23^
Doxycycline (tetracyclin)	Bacteriostatic	Ribosomal 30S and 50S subunits	Inhibition endotoxins^24^
Erythromycin (macrolide)	Bacteriostatic	Ribosomal 50S subunit	Virulence reduction^20^
Gentamicin (aminoglycoside)	Bactericidal	Ribosomal 30S subunit	Virulence reduction^20^ Poor biofilm reduction^25^
Vancomycin (glycopeptide)	Bactericidal	Inhibition cell wall synthesis	No effect on SaeRS^13^ Poor biofilm reduction^25^
Resveratrol (polyphenolic phytoalexin)	Bacteriostatic	Affects tyrosine tRNA	Downregulation SaeRS^19^

Sub-MIC antimicrobial pressure exists in tissue surrounding biomaterial implants because treatment by antimicrobials is hindered by poor penetration into the biofilm or by pathological changes in the implant site, like the formation of a fibrous layer around the implant in case of older implants (> 1 month)^26^, or changes to the bone structure in joint prosthesis^27^.

This study aimed at investigating the relation between biofilm formation and MN production and the impact of sub-MIC antimicrobial pressure on this relation. Therefore biofilm formation and MN activity in planktonic and biofilm cultures were studied while applying various sub-MICs of antimicrobials to three strains of *S. aureus*, the *S. aureus* ATCC12600, the bioluminescent *S. aureus* Newman lux, and the *S. aureus* Newman lux Δ*nuc1* mutant which is deficient in producing MN^17^. The ATCC12600 is often used as a reference strain. The Newman WT strain was chosen because of its constitutive expression of MN due to a point mutation SaeS^p^ that constitutively activates the response regulator SaeR, even in the early-exponential growth phase^28^. Five antibiotics were involved with different working mechanisms (Table 1) and one antimicrobial which has been shown to inhibit *SaeRS* activity^19^. Sub-MIC antimicrobial pressure, in particular by aminoglycocides, is also known to affect the production of PIA, the other main important biofilm apart from e-DNA, that contributes to biofilm formation and immune evasion mechanisms^29^. Thus, in order to take into account biofilm characteristics that may be affected by sub-MICs of antimicrobials in relation to MN activity, both total biomass and polysaccharide quantity in the EPS were investigated as well as colony forming units (CFU’s) and bioluminescence as a measure of metabolic activity^30^.

## Results

### CFU, polysaccharides and biomass of biofilms in absence of antimicrobials

**Figure 1:**
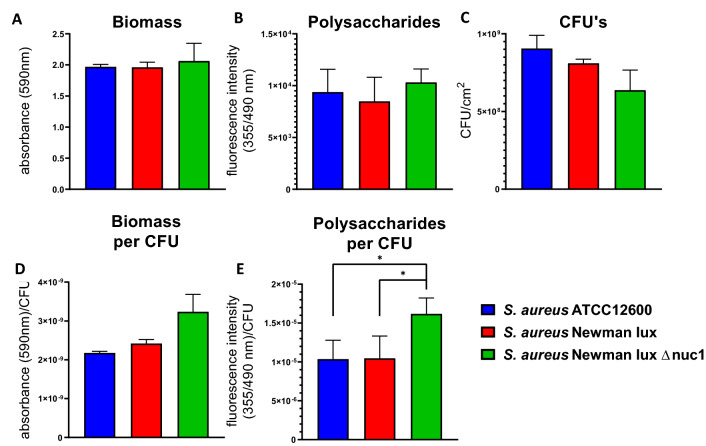
Biofilm characteristics of *S. aureus* ATCC12600, Newman lux, and Newman lux Δ*nuc1*. (**A**) Biomass of biofilms measured by spectral absorption after crystal violet staining. (**B**) Polysaccharide content of biofilms measured by fluorescent intensity after calcofluor white staining (**C**) Colony forming units (CFU’s) per cm^2^ of biofilms grown for 24 h in a 24 wells plate. (**D**) Ratios of biomass/CFU and (**E**) polysaccharides/CFU of biofilms. All data shown are averages of triplicate measurements in three separate cultures, repeated on separate days. Error bars indicate standard error of the mean. Statistical differences were calculated using an ANOVA. *Significant difference (p < 0.05). In (**D**, **E**) the natural logarithm of the values was used for statistical analysis as the data are lognormally distributed (see also Fig. S3).

The total biomass, polysaccharide content and CFUs of the two MN proficient strains and one MN deficient *S. aureus* strain after 24 h of biofilm growth were not significantly different (Fig. 1A–C) due to relatively large standard deviations encountered over all cultures and experiments for all parameters. The analysis, of biomass and polysaccharides per CFU however, executed within each separate culture and experiment on one and the same day revealed that biomass per CFU was raised in the MN deficient strain, although not significantly (Fig. 1D). However, the amount of polysaccharides produced per CFU of the MN deficient *S. aureus* Newman lux Δ*nuc1* grown under biofilm conditions was significantly higher than the other strains (Fig. 1E), indicating that an equally amount of EPS was produced populated by fewer viable bacteria. Note that biomass and polysaccharide content do not need to be directly proportional to each other, since biomass includes both bacterial mass (live and dead) and biofilm matrix.

### Changes in biofilm composition under antimicrobial pressure

As expected, most *S. aureus* biofilms required a higher concentration of antimicrobials to achieve inhibition of growth than planktonically grown bacteria resulting in MBIC > MIC (Table 2). The exceptions being doxycycline and erythromycin, for which no differences between planktonic and biofilm inhibition were observed for both Newman strains. It should be noted that the susceptibility to antimicrobial substances of the Newman *nuc1*-deficient strain was identical to its parent strain (Table 2).

**Table 2:** Resistance cutoff points^31^, minimal inhibitory concentrations (MIC) and minimal biofilm inhibitory concentrations (MBIC) (µg/ml) of antimicrobials for *Staphylococcus aureus* ATCC12600, Newman lux, and Newman lux Δnuc1 strains.

	Resistant when MIC≥	*S. aureus ATCC12600*		*S. aureus Newman lux*		*S. aureus Newman lux* Δ*nuc*	
		MIC	MBIC	MIC	MBIC	MIC	MBIC
*Ciprofloxacin*	4	1	2	8	16	8	16
*Doxycycline*	16	0.0625	0.5	0.125	0.125	0.125	0.125
*Erythromycin*	8	64	512	64	64	64	64
*Gentamicin*	16	2	8	1	2	1	2
*Vancomycin*	16	1	2	1	2	1	2
*Resveratrol*	Not used clinically	128	> 512	> 512	> 512	> 512	> 512

**Figure 2:**
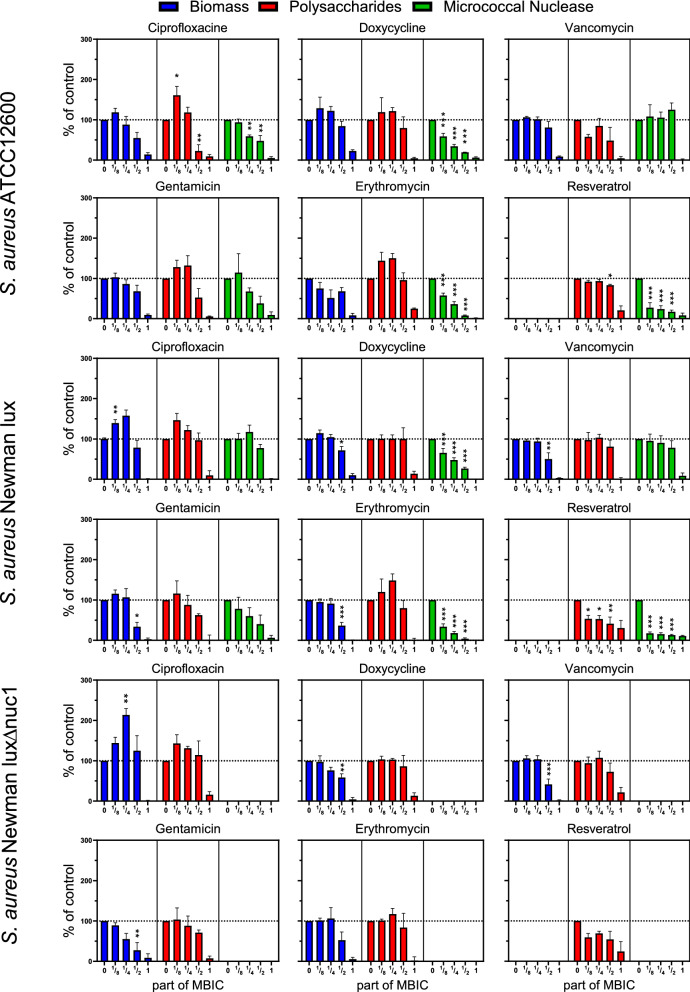
Biomass, polysaccharides and micrococcal nuclease (MN) activity of *S. aureus* ATCC12600, Newman lux, and Newman lux *Δnuc1* biofilms under antimicrobial pressure (at 0, 1/8, $${\raise0.7ex\hbox{$1$} \!\mathord{\left/ {\vphantom {1 4}}\right.\kern-\nulldelimiterspace} \!\lower0.7ex\hbox{$4$}}$$, $${\raise0.7ex\hbox{$1$} \!\mathord{\left/ {\vphantom {1 2}}\right.\kern-\nulldelimiterspace} \!\lower0.7ex\hbox{$2$}}$$ or 1 times MBIC). All data are normalized to a control culture without antimicrobials (= 100%; horizontal dotted line). No MBIC was found for resveratrol, therefore it is set at the highest attainable concentration, 512 μg/mL. Statistical differences were calculated using an ANOVA. *Significant difference for concentrations smaller than MBIC when compared to the culture without antibiotics. (*p < 0.05; **p < 0.01; ***p < 0.001).

Most sub-inhibitory concentrations of antimicrobials had no significant inhibiting effects on total biomass and polysaccharides (see Fig. 2). Ciprofloxacin had a stimulating effect on biomass at low doses ($${\raise0.7ex\hbox{$1$} \!\mathord{\left/ {\vphantom {1 8}}\right.\kern-\nulldelimiterspace} \!\lower0.7ex\hbox{$8$}}$$ and $${\raise0.7ex\hbox{$1$} \!\mathord{\left/ {\vphantom {1 4}}\right.\kern-\nulldelimiterspace} \!\lower0.7ex\hbox{$4$}}$$ MBIC) in both *S. aureus* Newman strains. Total MN activity of the biofilms was inhibited up to 80% by doxycycline, erythromycin and resveratrol at concentrations below the MBIC (See Fig. 2). At high concentrations (± 100 μg/mL) resveratrol gradually precipitated, binding crystal violet and causing a strong false positive signal. Precipitated resveratrol, however, had no effect on the quantification of MN activity and polysaccharides (data not shown).

No significant difference was found in biomass and polysaccharide between *S. aureus* Newman lux and the nuc1-deficient strain at any concentration of any antimicrobial (Fig. S1).

### Effects of antimicrobials on EPS production per bacterium

**Figure 3:**
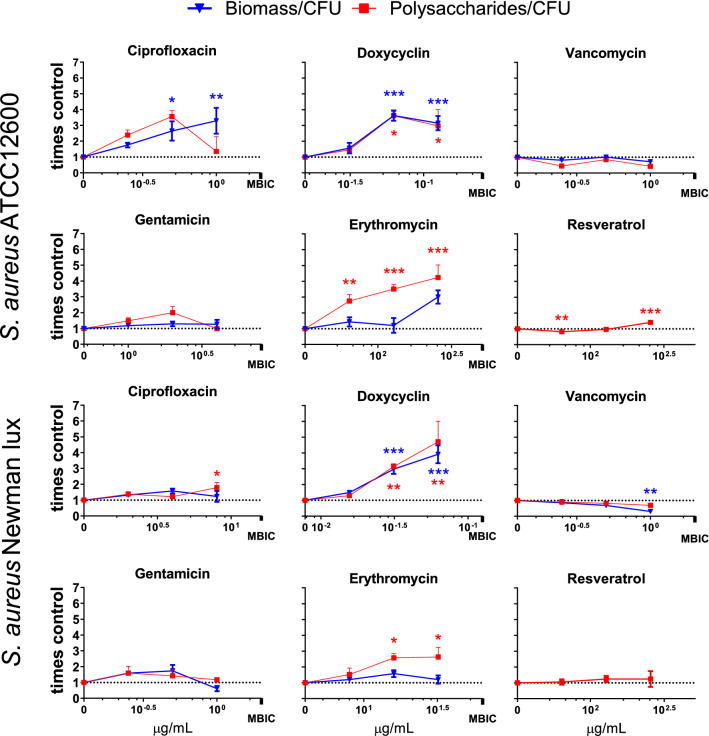
Change in biomass per CFU and polysaccharides per CFU ratios of *S. aureus* ATCC12600 and Newman lux biofilms. Biomass is quantified by crystal violet staining and subsequent measurement of spectral absorption at 590 nm. Polysaccharides are quantified by calcofluor white and subsequent measurement of fluorescent intensity at 355/490 nm (Ex/Em). Values shown is increase relative to the control biofilms without antibiotics (= 1; dotted horizontal line). Data shown are based on averages of triplicate measurements, repeated on separate days. Error bars indicate standard error of the mean. Statistical differences were calculated using an ANOVA comparing lognormal values (see “Materials and methods”). *Significant difference when compared to the culture without antibiotics (*p < 0.05; **p < 0.01; ***p < 0.001).

Some antimicrobials induced increase of EPS production per CFU under antimicrobial pressure was observed. Ciprofloxacin, doxycycline, and erythromycin increased the biomass and polysaccharides per CFU significantly by up to five times that of the control (Fig. 3), whereas gentamicin, vancomycin and resveratrol had a moderate to no effect. This is due to the biomass and polysaccharide content staying largely unchanged while the number of CFU’s decreased (Figs. 2 and S2).

### Effects of antimicrobials on nuclease activity per bacterium

**Figure 4:**
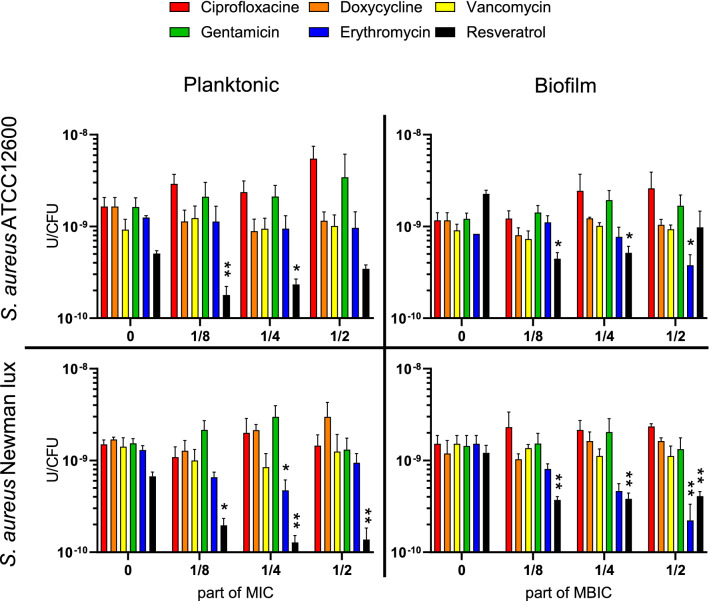
Micrococcal nuclease (MN) production per CFU of *S. aureus* ATCC12600 and Newman lux, growing planktonically and in biofilm under antibiotic pressure. Data shown are averages of triplicate measurements, repeated on separate days. Error bars indicate standard error of the mean. Statistical differences were calculated using an ANOVA comparing lognormal values with control values where antimicrobial concentration = 0). *Significant difference between value and the culture without antibiotics (*p < 0.05; **p < 0.01).

CFU-counts in both biofilm and planktonic cultures (Fig. S2) were used to calculate the effect of antimicrobials on MN activity per viable bacterium. In contrast to the stimulating effect of some antimicrobials on biomass and polysaccharides, MN activity per CFU was unaffected or inhibited by antimicrobials (Fig. 4). Lognormal values of MN/CFU were used because this variable was lognormally distributed (see Fig. S3). In particular resveratrol inhibited MN per CFU significantly at concentrations as low as $${\raise0.7ex\hbox{$1$} \!\mathord{\left/ {\vphantom {1 8}}\right.\kern-\nulldelimiterspace} \!\lower0.7ex\hbox{$8$}}$$ MIC and MBIC, except for *S. aureus* ATCC12600 biofilms in which MN per CFU was inhibited at $${\raise0.7ex\hbox{$1$} \!\mathord{\left/ {\vphantom {1 8}}\right.\kern-\nulldelimiterspace} \!\lower0.7ex\hbox{$8$}}$$ and $${\raise0.7ex\hbox{$1$} \!\mathord{\left/ {\vphantom {1 4}}\right.\kern-\nulldelimiterspace} \!\lower0.7ex\hbox{$4$}}$$, but not at $${\raise0.7ex\hbox{$1$} \!\mathord{\left/ {\vphantom {1 2}}\right.\kern-\nulldelimiterspace} \!\lower0.7ex\hbox{$2$}}$$ MBIC. Erythromycin reduced MN activity of the Newman strain by almost 75% at $${\raise0.7ex\hbox{$1$} \!\mathord{\left/ {\vphantom {1 4}}\right.\kern-\nulldelimiterspace} \!\lower0.7ex\hbox{$4$}}$$ MBIC.

### Effect of different type of culturing

**Figure 5:**
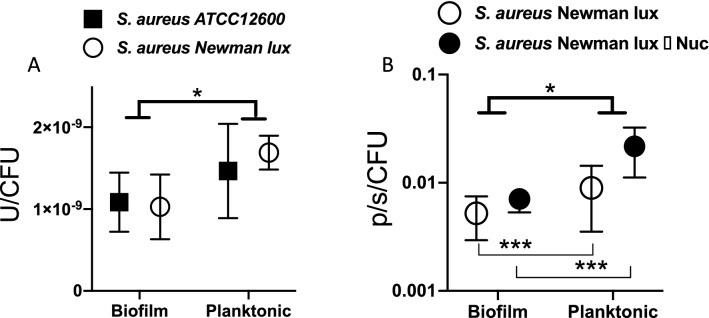
(**A**) Micrococcal nuclease activity (U), per colony forming unit (CFU) of *S. aureus* ATCC12600 and *S. aureus* Newman lux from planktonic and biofilm cultures. The type of culture (biofilm or planktonic) significantly affects the nuclease production. (**B**) Bacterial luminescence per CFU (photons/second/CFU) of *S. aureus* Newman lux and *Δnuc1* mutant. The type of culture (biofilm or planktonic) significantly affects the luminescence for both strains individually. Data shown are averages of triplicate measurements, repeated on separate days. Statistical analysis was done using an ANOVA accounting for strain and type of culture. Error bars indicate standard deviations. Asterisks indicate significant differences (*p < 0.05; ***p < 0.001).

Bacteria were grown both in stationary biofilm cultures from adhering bacteria, and under rotational movement to stimulate planktonic growth and reduce biofilm formation (see also Fig. S4). MN production and luminescence per CFU was quantified and analyzed using a two-way ANOVA. Lognormal values of MN/CFU were used as this variable is lognormally distributed (see Fig. S3). Planktonically grown bacteria produced more MN per CFU than those growing in a biofilm (p = 0.0118) (Fig. 5A). Strain was not a significant determining factor, neither was interaction between strain and type of culturing. As expected, the *nuc1*-deficient strain produced less than 1% of the amount MN produced by the parent strain. Luminescence per CFU in strains grown under rotation was 150% higher than when grown stationary (p = 0.0003) (Fig. 5B). In case of luminescence the deletion of the nuc1 gene was a significant factor in increasing luminescence (p = 0.0028), with a significant interaction between type of culturing and nuc1 deletion (p = 0.0219). The luminescence was used as a marker for metabolic activity of the bacteria^30^.

## Discussion

The aim of this study was to investigate the relation between biofilm formation and MN activity and the impact on it by sub-MIC antimicrobial pressure.

Various earlier reports are not unambiguous on whether MN has an inhibitory effect on biofilm formation with results varying from increased to decreased biofilm formation, also depending on in vitro^17^ or in vivo setup^11, 17, 18, 32^. Our results revealed no significant differences between the two WT-strains and the MN-deficient strain with respect to the number of CFU, the total biomass and polysaccharides (Figs. 1A–C and S1). It might have been anticipated that *S. aureus* Newman strains do not produce biofilms due to a defect in the expressed fibronectin binding proteins (FnBPs) but it has been suggested earlier that the lack of proper fibronectin binding proteins is compensated by the enhanced production of extracellular adhesive proteins (Eap) in the Newman strain^33^. Forson et al. also suggested that the eDNA in the biofilm is stabilized, possibly via interactions with DNA-binding proteins like Eap and in particular SaeP, which is expressed as an auxiliary protein in the *SaeRS* two component system^32^. There are several possible explanations for the observation that biofilms in our experiments did not increase in mass upon deletion of the *nuc1* gene as was observed in other research^17^, such as the lack of added glucose in the growth medium, longer incubation time (24 h), and the lower initial bacterial load that may alter the response of the biofilm to MN^11, 34, 35^.

When the present results, however, were normalized with respect to the number of CFU’s in the assays, the results revealed, for the first time, a significant, 20% enhancement of biomass and polysaccharides production per CFU of the *nuc1* deficient *S. aureus* Newman lux strain compared to both other *S. aureus* strains. This suggests an inverse relationship between EPS production per CFU and MN activity per CFU. This is in line with earlier findings^32^, based on bacterial counts and optical coherence tomography observations, that the bacterial density in biofilms is reduced in the nuclease deficient *S. aureus* Newman strain. Uncleaved eDNA in the nuclease deficient strain tends to expand the biofilm and may keep bacteria at relatively long distances from each other, also pointing to an increased biomass production per CFU in MN deficient strains^32^. It is very likely that the differences found between both Newman strains are due to the missing *nuc1* gene alone and not to other secondary mutations in regulatory genes. Both Newman strains showed very similar adhesion characteristics on both hydrophobic and hydrophilic substrates and were also highly similar in biofilm structure^32^. Moreover, both strains after inoculation in mice to induce a local infection in the murine thigh, resulted in similar infections as observed by in vivo imaging^36^.

In this study we anticipated that some antibiotics may modulate MN production, the most significantly in *the S.aureus* ATCC12600 strain, since the constitutive character of the SaeS^p^ induced kinase activity. The point mutation in the Newman strain expression, however, allows some substances (such as a biocide “perform” and Sodium dodecyl sulfate) to raise the transcription level of SaeS even further^33^ indicating that environmental factors may still modulate the Sae system and potentially modulate nuclease production as remarkably found in our study in both *S. aureus* strains. We observed significant reductions, up to 80% in MN production for both *S. aureus* strains by doxycyclin, erythromycin and resveratrol by (Fig. 2). The inhibition of MN activity by erythromycin is in line with reports of clindamycin, which has a similar working mechanism by binding to the ribosomal 50 s subunit and inhibits the *SaeRS* system (Table 1). The reduction by resveratrol is attributed to the direct inhibition of the *SaeRS* system as reported earlier^19^*.* Since it was concluded from Fig. 1E that MN reduction had effect on the EPS production per CFU rather than on the total CFU count (Fig. 1C), biomass (Fig. 1A) and polysaccharides (Fig. 1B), it was expected that EPS production per CFU subsequently would be affected by sub-MIC pressure of antimicrobials as well. Indeed, significant higher polysaccharide content per CFU was found in the presence of sub-MIC doxycyclin and erythromycin, up to a factor of 5 with respect to the control (Fig. 3), but only in case of erythromycin this coincides with significant reduction in MN per CFU (Fig. 4) and not in case of sub-MIC doxycyclin. These results strongly suggest that sub-MIC doxycyclin stimulates polysaccharide production, an effect that has been reported earlier for aminoglycosides in *S. epidermidis*^29^. The aminoglycoside in this study (gentamycin), however, did not show polysaccharide stimulation.

It should be noted that as a result of the highly increased histidine kinase activity in the Newman strain, we expected that *S. aureus* ATCC12600 would show a lower nuclease productivity. Instead, we found relatively high amounts of nuclease produced by the *S. aureus* ATCC12600, in the same range as the Newman strain (see Fig. 5A). We earlier identified *S. lugdunensis* as a additional strain producing an equal amount of nuclease, and *S. aureus* RN 4220 hardly showed lower nuclease production as compared to the Newman strain^16^. Moreover it should be noticed that the Newman *SaeS*^*p*^ activates SaeR in the early exponential phase, resulting in an early *nuc1* mediated reaction^28, 37–39^. The ‘normal’ *SaeS* found in ATCC12600 activates *SaeR* only after 6 h of growth. Since our measurements have all been made after 24 h of growth this difference may have disappeared entirely, since earlier experiments have shown that nuclease production of strains usually levels to a plateau value after 12 h of growth^16^. As was observed earlier^32^, MN activity appeared significantly inhibited in the biofilm mode of growth compared to planktonic growth (Fig. 5A). The reduction of MN in biofilms compared to planktonic cultures, is similar to the reduction of bioluminescence in biofilms as related to planktonic cultures (Fig. 5B). The reduction in bioluminescence most likely results from a limited availability of oxygen and lower metabolic activity of bacteria located deeper in the biofilm^30, 40^. Decreased oxygenation and metabolic activity is probably also the dominant factor reducing MN production in biofilms.

We recognize a wide debate on the various mechanisms involved in biofilm formation and we in particular elaborated on the understudied aspects of MN production and its potential impact on biofilm characteristics. In summary our results do not confirm earlier findings that MN directly influences total biomass of the biofilm, but rather that deletion of *nuc1* stimulates the EPS production per CFU, as was found in the *S. aureus* Newman strain. MN production can be further modulated by antimicrobial pressure, and did in some cases coincide with significantly increased production of polysaccharides, but this was not a consistent relationship and might be a result of other more complex regulatory mechanisms. It is suggested that various antibiotics differentially impact the delicate balance of eDNA release and nuclease production. Unraveling the underlying mechanisms, however, was not the primary objective of this study, rather investigating the effect of antibiotics on nuclease and biofilm formation as it has clinical relevance by various reasons. First sub-MIC antibiotic pressure develops around biomaterial implants during antibiotic treatment. Second, modulation of MN production of *S. aureus* may result in clinically relevant strategies of preventing and treating *S. aureus* infections by arresting biofilm dispersal. Finally, *S. aureus* bacteria possess escape mechanisms from the host immune system in particular by MN that disassembles neutrophil extracellular traps, rendering the bacteria unaffected by this particular defense mechanism of the immune system^41, 42^. Reduction of MN by sub-MIC antimicrobials may thus assist in the eradication of biofilms by the host immune system, even in case of clinical resistance to these antimicrobials.

## Materials and methods

### Antimicrobials

The antimicrobials used are mentioned in Table 1 together with their working mechanism. They were purchased from Merck (Darmstadt, Germany). All antimicrobials were prepared from powder-form in ultrapure water and sterilized by filtering through a 0.22 μm pore filter according to the manufacturers protocol. Resveratrol was dissolved in DMSO (dimethylsulfoxide) to a concentration of 52 mg/ml before filtration. Due to the poor aqueous solubility of resveratrol no concentrations of resveratrol higher than 512 µg/ml in ultrapure water containing 1% DMSO were used. All cultures containing resveratrol including the control for these cultures without antibiotics, contain 1% DMSO.

### Nuclease probe

In order to measure MN production a nuclease activatable fluorescence probe was applied^16, 36^. The probe (Merck) consists of a 11-thymine base oligonucleotide flanked by a fluorescein amidine fluorophore and both a ZEN and Black hole quencher (5′-/FAM/TTTTTTTTTTT/ZEN/BhQsp/-3′). Before use the lyophilized probe was diluted in 10 mM Tris–HCl and 10 mM CaCl_2_, pH 8.0 to a working stock concentration of 2 µM.

### Bacterial strains, culturing and harvesting

Experiments were conducted with *S. aureus* ATCC12600 and a luminescent *S. aureus* Newman lux strain (AH2600) in which LuxABCDE genes and kanamycin resistance were transduced from *Photorhabdus luminescens* using bacteriophage 11^43^. The bioluminescence is the result of a LuxABCDE gene, regarded as a global marker for cellular activity. The *S. aureus* Newman lux Δnuc1 mutant strain (AH2627) was obtained by deletion of *nuc1* using the Targetron Gene Knockout System (Merck)^17^. Both *S. aureus* Newman strains were constructed previously^17, 36^.

All media were prepared according to manufacturer’s protocol. *S. aureus* ATCC12600, *S. aureus* Newman lux and *S. aureus* Newman lux Δnuc1 were cultured from cryopreservative beads onto Tryptic Soy Broth Agar (TSA) (Oxoid, Basingstoke, UK). Kanamycin (200 μg/ml) was added to the agar plates, pre-, and main-cultures of the *S. aureus* Newman lux strains. After inoculation the agar plates were incubated for 24 h at 37 °C in ambient air.

A pre-culture was made by inoculating one colony in tryptic soy broth (TSB) (10 ml) (Oxoid) and cultured for 24 h at 37 °C, 150 RPM. The main culture was made by inoculating 40 ml TSB with 2 ml of the pre-culture and cultured for 16 h at 37 °C, 150 RPM.

The bacteria were harvested by centrifugation for 5 min at 10 °C, 5000*g* (Avanti J-E centrifuge, JLA-16.259 rotor, Beckman-Coulter) and subsequently washed three times with phosphate buffered saline (PBS). All cultures were sonicated three times for 10 s at 30 W on ice, to remove aggregates. The number of bacteria was determined from a 1:200 diluted sample in a Bürker-Türk counting chamber in order to establish the required concentration of bacteria to start the experiments.

### Minimum inhibitory concentration and minimum biofilm inhibitory concentration

The MIC of all antimicrobials were determined by incubating all precultured strains for 24 h at 37 °C in a 1:1 serially diluted antimicrobial concentration under rotating conditions (150 RPM) starting from 512 µg/ml. To this end all wells but the first were filled with 100 μl of ultrapure water. Then an antimicrobial solution (200 μl) at double the final concentration (1024 µg/ml in this case) was added to the first well. Subsequently 100 μl was transferred from the first well to the next, and then from that one to the next etc. Then a suspension of double the final concentration of bacteria (2 × 10^5^ cells/mL in this case) in double concentrated TSB was prepared, and 100 μl was added to all wells, resulting in all wells containing the final concentration of bacterial inoculum in TSB, with an antimicrobial gradient. Wells with sterile TSB were included as negative controls. After 24 h the plates were assessed for the lowest concentration that resulted in no visible growth (MIC).

To determine the minimum biofilm inhibitory concentration (MBIC), wells in a flat-bottom 96 wells plate (Greiner Bio-One) were filled with 200 μl of 5 × 10^8^ bacteria/ml in TSB. Bacteria were allowed to adhere for 1 h at 37 °C under stationary conditions. Non-adhering bacteria were removed by washing three times with PBS before addition of an antimicrobial gradient as described above, followed by stationary incubation for 24 h at 37 °C. Biomass was then assessed by staining with crystal violet and spectral absorption. To assess the biomass, the biofilms were washed gently three times with PBS and dried at 60 °C to fix the biofilms. Biofilms were stained for 5 min with crystal violet (0.06%) and washed three times with demineralized water^44^. Subsequently, crystal violet was resolubilized in 30% acetic acid for at least 15 min. Finally, the crystal violet solution was diluted four times and the absorbance was measured at 590 nm in a Fluostar Optima plate reader (BMG labtech, Ortenberg, Germany). This device is able to measure absorbance up to a value of 4.5. Values measured across all experiments did not exceed 50% of this maximum. MBIC was defined as a ≥ 90% inhibition of biofilm formation^45^.

### Effect of antimicrobials on planktonic cultures

All planktonic cultures were grown in a 24 (polystyrene) well plate (Greiner Bio-One, Frickenhausen, Germany) with a final volume of 1120 μl per well with decreasing antimicrobial concentrations, established by a 1:1 serial dilution ranging from 4 times to 1/16 times the MIC as described above with adjusted volumes. Wells with sterile TSB were included as negative controls. The final volume of 1120 μl for each well was chosen to preserve the volume-to-surface ratio across all experiments. 1% DMSO was added to the wells that served as a control for the strains grown with resveratrol. The concentration of bacteria at T = 0 was 1 × 10^5^ bacteria/ml. All cultures were grown as described under MIC and MBIC.

For each of the concentrations of antimicrobials the number of CFU’s and the production of MN were determined in the same bacterial culture in order to allow the calculation of MN production per CFU.

### Effect of antimicrobials on biofilm cultures

Biofilms were grown as described above. The differential concentrations of antibiotics were established by a 1:1 serial dilution that ranged from 1 time to 1/8 times MBIC. 1% of DMSO was added to the wells that served as a control for the strains grown with resveratrol. Wells with sterile TSB were included as negative controls. Separate biofilm cultures were prepared for measuring biofilm biomass, polysaccharide quantification, and CFU count/MN production. The former two were quantified from a single biofilm culture in order to allow the calculation of nuclease production per CFU. In order to relate biofilm formation at various antimicrobial concentrations, biofilms of all strains were grown simultaneously on the same day for all concentrations.

### Determination of biomass and polysaccharides

To assess the biomass of the biofilm the same crystal violet procedure was followed as described for the determination of the MIC and MBIC. To quantify the polysaccharides 100 μl medium was carefully removed from each well containing a biofilm and replaced by 100 μl calcofluor white (Sigma Aldrich) solution (40 μg/ml) to yield a final concentration of 20 μg/ml calcofluor white per well which binds to polysaccharides^44^. After 1 min all wells were gently washed three times with PBS. Biofilms were resuspended by pipetting forcefully up and down and 5 min of sonication of the plate in a sonication bath. Fluorescence was measured in a Fluostar Optima plate reader (Excitation: 355 nm/Emission: 490 nm, setting: bottom).

### Determination of CFU’s

All biofilms were resuspended by pipetting forcefully up and down and 5 min of sonication of the plate in a sonication bath. From the 24-h planktonic cultures a sample was taken directly from the suspension. The samples (20 μl) were serially tenfold diluted in PBS (180 μl). Three 10 μl aliquots of every dilution (10–10^7^ times diluted) were put on a TSA plate and grown for 18 h at 37 °C. Then the number of colonies were counted, and the number of CFU per cm^2^ (biofilms) or per mL (planktonic) was calculated.

### Nuclease activity

Bacterial suspensions of resuspended biofilm and planktonic cultures were obtained from the same cultures as from which the samples for CFU counting were taken. To measure nuclease activity samples were diluted 1000 times with 10 mM Tris–HCl, 10 mM CaCl_2,_ pH 8.0 buffer. Twenty-five µl of diluted bacterial suspension was combined with 25 µl of probe working stock (2 µM) and 10 mM Tris–HCl, 10 mM CaCl_2,_ pH 8.0 (150 µl), buffer in a 96 wells plate. As a negative control 1:1000 diluted sterile TSB was used instead of a sample from the culture. Fluorescence intensity was measured with a Fluostar Optima plate reader (Excitation: 490 nm; Emission: 520 nm) at 1-min intervals for 5 min at 37 °C. Nuclease activity was determined by the rate of fluorescence change per min. We used a known amount of purified MN (Merck) to calibrate the nuclease probe. The rate of fluorescence per min was shown to be linear with MN concentration (Fig. S5). One unit (U) is defined as the amount of enzyme required to release acid soluble oligonucleotides that produce an absorbance increase of O.D. 1.0 at 260 nm in 30 min at 37 °C, pH 8.8^46^.

### Bioluminescence

To quantify bioluminescence, plates containing the biofilm or planktonic cultures were placed in an IVIS Lumina 2 system (PerkinElmer, Waltham Massachusetts US) and imaged for one minute (excitation filter: blocked, emission filter: open). Data was analyzed using the LivingImages 4.7.2 software (PerkinElmer). Reported units are in photons per second leaving the entire volume of the well.

### Statistical analysis

All data were analyzed using Graphpad Prism 9 (Graphpad, San Diego, United States). Difference between 2 groups was calculated using a T-test, while differences between more groups were calculated using an ANOVA where appropriate. All statistics concerning MN/CFU, biomass/CFU, and polysaccharides/CFU were based on log-normal values, as these values are lognormally distributed and its log-values fit a normal distribution (Fig. S3). All experiments were performed in triplicate on each of three separate days with different cultures. Reported values are average values over nine measurements, standard deviations, standard errors of the mean and statistical tests are based on the three average values obtained, one for each culture.

## Supplementary Information

Supplementary Figures.

## References

[1] Busscher HJ (2012). Biomaterial-associated infection: Locating the finish line in the race for the surface. Sci. Transl. Med..

[2] O’Toole P, Maltenfort MG, Chen AF, Parvizi J (2016). Projected increase in periprosthetic joint infections secondary to rise in diabetes and obesity. J. Arthropl..

[3] Voigt A, Shalaby A, Saba S (2010). Continued rise in rates of cardiovascular implantable electronic device infections in the United States: Temporal trends and causative insights. PACE Pacing Clin. Electrophysiol..

[4] Conlon BP (2014). *Staphylococcus aureus* chronic and relapsing infections: Evidence of a role for persister cells: An investigation of persister cells, their formation and their role in *S. aureus* disease. BioEssays.

[5] Flemming HC (2016). Biofilms: An emergent form of bacterial life. Nat. Rev. Microbiol..

[6] Moormeier DE, Bose JL, Horswill AR, Bayles KW (2014). Temporal and stochastic control of *Staphylococcus aureus* biofilm development. MBio.

[7] Peterson BW, Van der Mei HC, Sjollema J, Busscher HJ, Sharma PK (2013). A distinguishable role of eDNA in the viscoelastic relaxation of biofilms. MBio.

[8] Dengler V, Foulston L, DeFrancesco AS, Losick R (2015). An electrostatic net model for the role of extracellular DNA in biofilm formation by *Staphylococcus aureus*. J. Bacteriol..

[9] Arciola CR, An YH, Campoccia D, Donati ME, Montanaro L (2005). Etiology of implant orthopedic infections: A survey on 1027 clinical isolates. Int. J. Artif. Organs.

[10] Hu Y, Xie Y, Tang J, Shi X (2012). Comparative expression analysis of two thermostable nuclease genes in *Staphylococcus aureus*. Foodborne Pathog. Dis..

[11] Mann EE (2009). Modulation of eDNA release and degradation affects *Staphylococcus aureus* biofilm maturation. PLoS ONE.

[12] Kavanaugh JS (2019). Identification of extracellular DNA-binding proteins in the biofilm matrix. MBio.

[13] Geiger T, Goerke C, Mainiero M, Kraus D, Wolz C (2008). The virulence regulator sae of *Staphylococcus aureus*: Promoter activities and response to phagocytosis-related signals. J. Bacteriol..

[14] Davis A, Moore IB, Parker DS, Taniuchi H (1977). Nuclease B. A possible precursor of nuclease A, an extracellular nuclease of *Staphylococcus aureus*. J. Biol. Chem..

[15] Kiedrowski MR (2014). *Staphylococcus aureus* Nuc2 is a functional, surface-attached extracellular nuclease. PLoS One.

[16] Rosman CWK (2018). ex vivo tracer efficacy in optical imaging of *Staphylococcus aureus* nuclease activity. Sci. Rep..

[17] Kiedrowski MR (2011). Nuclease modulates biofilm formation in community-associated methicillin-resistant staphylococcus aureus. PLoS ONE.

[18] Beenken KE, Spencer H, Griffin LM, Smeltzer MS (2012). Impact of extracellular nuclease production on the biofilm phenotype of *Staphylococcus aureus* under In vitro and In vivo conditions. Infect. Immun..

[19] Duan J (2018). Subinhibitory concentrations of resveratrol reduce alpha-hemolysin production in *Staphylococcus aureus* isolates by downregulating saeRS. Emerg. Microbes Infect..

[20] Hodille E (2017). The role of antibiotics in modulating virulence in *Staphylococcus aureus*. Clin. Microbiol. Rev..

[21] Kaplan JB (2012). Low levels of β-lactam antibiotics induce extracellular DNA release and biofilm formation in *Staphylococcus aureus*. MBio.

[22] Mashruwala AA, Gries CM, Scherr TD, Kielian T, Boyd JM (2017). SaeRS is responsive to cellular respiratory status and regulates *Staphylococcus aureus*. Infect. Immun..

[23] Bisognano C (2004). A RecA-LexA-dependent pathway mediates ciprofloxacin-induced fibronectin binding in *Staphylococcus aureus*. J. Biol. Chem..

[24] Chopra I, Roberts M (2001). Tetracycline antibiotics: Mode of action, applications, molecular biology, and epidemiology of bacterial resistance. Microbiol. Mol. Biol. Rev..

[25] Ferrer MD (2017). Effect of antibiotics on biofilm inhibition and induction measured by real-time cell analysis. J. Appl. Microbiol..

[26] Bergan T (1981). Pharmacokinetics of tissue penetration of antibiotics. Rev. Infect. Dis..

[27] Jensen LK (2017). Suppurative inflammation and local tissue destruction reduce the penetration of cefuroxime to infected bone implant cavities. J. Comp. Pathol..

[28] Cue D (2015). SaeRS-dependent inhibition of biofilm formation in *Staphylococcus aureus* Newman. PLoS ONE.

[29] Nguyen HTT, Nguyen TH, Otto M (2020). The staphylococcal exopolysaccharide PIA—Biosynthesis and role in biofilm formation, colonization, and infection. Comput. Struct. Biotechnol. J..

[30] Daghighi S (2015). Influence of antibiotic pressure on bacterial bioluminescence, with emphasis on *Staphylococcus aureus*. Int. J. Antimicrob. Agents.

[31] The Clinical and Laboratory Standards Institute (2016). Performance Standards for Antimicrobial Susceptibility Testing CLSI supplement M100S.

[32] Forson AM, van der Mei HC, Sjollema J (2020). Impact of solid surface hydrophobicity and micrococcal nuclease production on *Staphylococcus aureus* Newman biofilms. Sci. Rep..

[33] Schäfer D (2009). A point mutation in the sensor histidine kinase saeS of *Staphylococcus aureus* strain Newman alters the response to biocide exposure. J. Bacteriol..

[34] Lovati AB, Bottagisio M, De Vecchi E, Gallazzi E, Drago L (2017). Animal models of implant-related low-grade infections. A twenty-year review. Adv. Exp. Med. Biol..

[35] Vidlak D, Kielian T (2016). Infectious dose dictates the host response during *Staphylococcus aureus* orthopedic-implant biofilm infection. Infect. Immun..

[36] Hernandez FJ (2014). Noninvasive imaging of *Staphylococcus aureus* infections with a nuclease-activated probe. Nat. Med..

[37] Mainiero M (2010). Differential target gene activation by the *Staphylococcus aureus* two-component system saeRS. J. Bacteriol..

[38] Jeong DW (2011). Identification of the P3 promoter and distinct roles of the two promoters of the SaeRS two-component system *in Staphylococcus au*reus. J. Bacteriol..

[39] Beenken KE (2014). Impact of the functional status of saeRS on in vivo phenotypes of *Staphylococcus aureus* sarA mutants. Mol. Microbiol..

[40] Sjollema J (2010). The potential for bio-optical imaging of biomaterial-associated infection in vivo. Biomaterials.

[41] Schilcher K (2014). Increased neutrophil extracellular trap-mediated *Staphylococcus aureus* clearance through inhibition of nuclease activity by clindamycin and immunoglobulin. J. Infect. Dis..

[42] Berends ETM (2010). Nuclease expression by *Staphylococcus aureus* facilitates escape from neutrophil extracellular traps. J. Innate Immun..

[43] Novick RP (1991). Genetic systems in Staphylococci. Methods Enzymol..

[44] Stiefel P (2016). Is biofilm removal properly assessed? Comparison of different quantification methods in a 96-well plate system. Appl. Microbiol. Biotechnol..

[45] Moskowitz SM, Foster JM, Emerson J, Burns JL (2004). Clinically feasible biofilm susceptibility assay for isolates of *Pseudomonas aeruginosa* from patients with cystic fibrosis. J. Clin. Microbiol..

[46] Heins JN, Suriano JR, Anfinsen B (1967). Characterization of a nuclease produced by *Staphylococcus aureus*. J. Biol. Chem..

