# Supplementary material for: Influence of sub-inhibitory concentrations of antimicrobials on micrococcal nuclease and biofilm formation in Staphylococcus aureus
Source: Sci Rep. 2021 Jun 24;11:13241. doi: 10.1038/s41598-021-92619-9 (PMC8225913; doi:10.1038/s41598-021-92619-9)
Supplement: Supplementary file 1 — Supplementary Figures. [file 41598_2021_92619_MOESM1_ESM.docx]

**Influence of sub-inhibitory concentrations of antimicrobials on micrococcal nuclease and biofilm formation in *Staphylococcus aureus***

Colin W.K. Rosman, Henny C. van der Mei, Jelmer Sjollema

*Supplemental material*

University of Groningen, University Medical Center Groningen, Department of Biomedical Engineering, Groningen, The Netherlands

Address correspondence to Jelmer Sjollema, j.sjollema@umcg.nl

**Figure S1.** Difference in total biomass and polysaccharide content between the *Staphylococcus aureus* Newman Δnuc1 mutant and its parent strain. Units on y-axis are relative differences (in percent) as related to the average difference between both strains grown in absence of antibiotics (control). Data shown are 3 averages of triplicate measurements, error bars indicate standard error of the mean. Each experiment had its own control. No significant differences were found.

Figure S2. *S. aureus* ATCC12600 and Newman lux grown under planktonic and biofilm conditions after 24 h under antimicrobial pressure. Biofilm growth is expressed in CFU/cm^2^, planktonic growth in CFU/ml. MIC and MBIC are marked with vertical dotted lines. Data shown are averages of triplicate measurements of separately cultured bacteria, error bars indicate standard error of the mean.


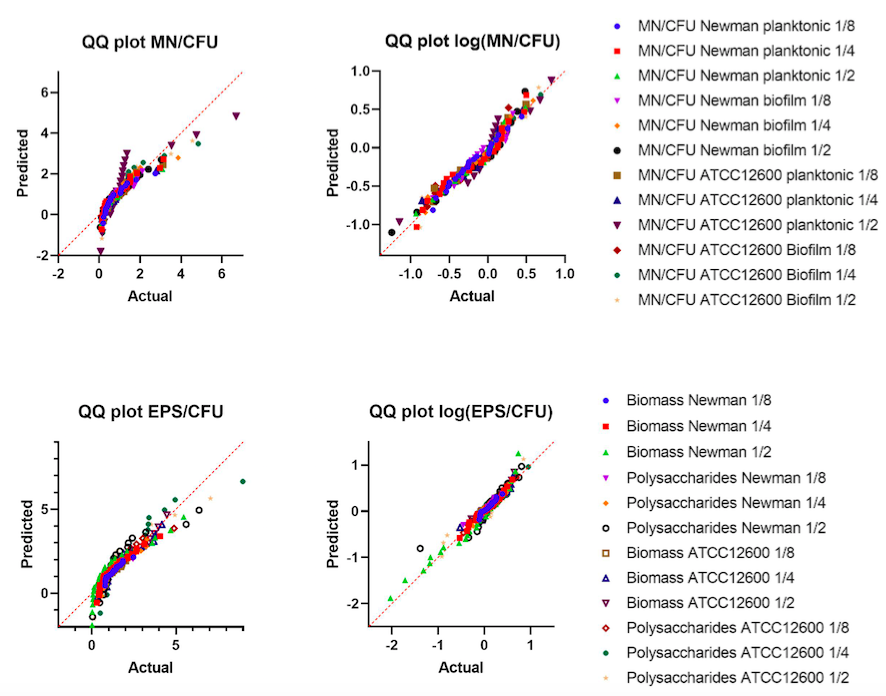


**Figure S3.**  Quantile-Quantile (QQ) plots of micrococcal nuclease per colony forming unit of both planktonic and biofilm mode of growth, and biofilm parameters biomass and polysaccharides (summarized as extracellular polymeric substance (EPS)) for both Staphylococcus aureus ATCC12600 and Newman (lux). Data on the horizontal axes are quantiles of actual distributions (or from distributions of log-value data), data on the y-axis represent quantiles on a theoretical normal distribution (or log-normal distribution). The graph shows that the distribution of data can be described by a log-normal distribution (both Figures on the right side of the panel) rather than by a normal distribution (Left part of the panel). Fractions in the legend refer to fraction of inhibitory concentration of all antimicrobials used.

**Figure S4.** Biomass of *S. aureus* Newman lux biofilm cultured statically and under flow (150 RPM). Values shown are separate cultures grown parallel on the same day. Horizontal bar and error bars indicate mean and 95% CI. The statistical difference was calculated using a t-test (***: p ≤ 0.001)

**Figure S5.** Calibration of the nuclease probe with micrococcal nuclease (MN). Graph showing the linear correlation (dotted lines = 95% CI) between fluorescence intensity increase per minute of the nuclease probe versus the quantity of the probe in U/mL. Due to photobleaching of the probe the fluorescence increase without MN is negative. Vertical dotted lines indicate the 5^th^ and 95^th^ percentile of all MN activities measures, excluding cultures with an inhibitory, or higher, concentration of antibiotics.
